# Editorial: Exploring oncolytic virus-mediated changes in immune microenvironment and immune cells in solid tumors

**DOI:** 10.3389/fimmu.2023.1234957

**Published:** 2023-09-19

**Authors:** Hongwei Zhang

**Affiliations:** Department of Neurosurgery, Sanbo Brain Hospital, Capital Medical University, Beijing, China

**Keywords:** oncolytic virus, cancer treatment, immune microenviroment, immune therapy, solid tumor

Oncolytic virus (OV) therapy is a promising cancer treatment strategy, which selectively replicates in and destroys tumor cells rather than normal cells, thereby effectively releasing viral PAMPs, molecular pattern signals, and cytokines into the tumor immune microenvironment, promoting the maturation of antigen-presenting cells, and activating antigen-specific CD4+ and CD8+ T cell responses. This series of reactions initiates potent antitumor responses and mediates tumor regression at distant tumor sites not exposed to the virus. OV therapy has achieved promising results in research studies with various solid tumors such as breast cancer, lung cancer, and ovarian cancer, suggesting that emerging OV cancer-targeting strategies are worthy of consideration as a promising complement to classical therapies.

Looking back on the history of OVs, since the mid-19th century, there have been reports of tumor patients infected by natural viruses in whom tumors shrank or even disappeared (1). Since then, the idea of using viruses to treat tumors has gained increasing attention, motivating more in-depth research. With the emergence of molecular technologies in the middle of the 20th century, researchers began to conduct *in vivo* experiments and clinical trials with OVs (2, 3). Multiple studies reported the killing effect of OVs on tumors (4–6). Research on OVs expanded, and more research results were published. In the 1990s, DNA recombination technology became standard, and engineering of OVs was carried out on a large scale. The pathogenicity of OVs was greatly reduced, and their oncolytic efficacy and tumor specificity were further enhanced. After a series of studies, in 2005 China approved the first human OV for tumor treatment, which has provided great inspiration for OV treatment research around the world (7).

In recent years, research reports on OV have increased rapidly, but these have not been summarized, and the changes in complex immune microenvironments and immune cells caused by OV treatment are still not fully understood. Therefore, this Research Topic focuses on the oncolytic virus-mediated immune microenvironment and immune cell changes in solid tumors with the goals of summarizing and evaluating the impact of OVs on the immune microenvironment and immune cells of solid tumors and identifying opportunities to further improve OV safety and efficacy. Oncolytic vaccinia virus (OVV) is one of the three OVs currently in common use. Sun Haijun et al. carried out a bibliometric screen of OVV studies published in English over the past 20 years and present their resulting detailed analysis of the development and evolution of OV therapy (Bo et al.).

More recent relevant clinical research on OV therapy alone or combined with other treatments considers use of OV therapy as a supplement or complement to more established treatments. A-Rum Yoon et al. describe in detail research progress on use of OVs as new cancer immunotherapy drugs, especially oncolytic adenovirus (OAd), oncolytic herpes simplex virus (OHSV) and OVV, and put forward their own views on the problems existing in the current OV clinical trials and the challenges that need to be overcome, especially for further improvements in efficacy (Yun et al.). Previous studies have demonstrated that OV therapy for tumors relies on a variety of mechanisms. In addition to a direct viral role in lysing tumor cells, cell death caused by an OV infection importantly fully exposes the immunogenicity of tumors, a process known as immunogenic cell death (ICD). ICD is an extremely complex process that activates innate and adaptive immunity through multiple pathways, resulting in a significant killing effect on tumors. The article by Liang-Tzung Lin et al. introduces in detail our current views on the mechanism of ICD caused by OV treatment and put forward their own opinions on how to regulate the immunogenicity of OV and enhance the therapeutic potential in cancer immunotherapy, especially through the application genome editing and combined use of ICD enhancers (Palanivelu et al.).

Due to the amenability of OVs to gene engineering, a large number of them have been modified to improve their stimulating effect on the immune system and thereby exert better immune-driven tumor killing. Jie Dong et al. edited the OVV to express bispecific T-cell engager (VV-BiTE) to build a bridge between tumor cells and T cells, making the immune response more intense and effective. The VV-BiTE, has shown a good anti-tumor effect and provided strong preclinical data for application in solid tumors (Wei et al.).

In addition to direct oncolytic effects, OVs can also play an important role as carriers for targeted gene therapy. Jonathan Blay et al. used bacteriophages as tumor-targeting vectors by transferring EGF genes into bacteriophage genes through genome editing technology so that the EGF polypeptide expression exists in their capsid, and then demonstrated that this bacteriophage can be continuously ingested by EGF ligand positive tumor cells, proving that it can play a role as an effective tool for gene therapy (Huh et al.). For the study of OV combined with ICD enhancers, an important current limitation is the lack of hamster immune checkpoint inhibitor (ICI) preparations. The mouse is the most commonly used preclinical animal model for evaluating the effectiveness of combination immunotherapy methods, but it does not support the replication of several human viruses. Therefore, the study of OV often uses a hamster model, which allows the replication of human adenovirus. However, the lack of hamster ICI preparations has been a significant limitation for research of oncolytic virus-ICI combination therapies. To address this problem, Akseli Hemminki et al. successfully produced the first Syrian hamster specific anti-PD-L1 monoclonal antibody, 11B12-1, solving the problem of the lack of ICI inhibitors for hamster models, and confirmed that the combination of OV TILT-123 and hamster anti-PD-L1 monoclonal antibody improved tumor growth control further compared to monotherapy, providing a powerful tool for further preclinical research of OV combination treatments *in vivo*(Club et al.).

As reflected in these reports, many new achievements have emerged in OV research, yielding further progress in applying OV therapy to change the immune microenvironment of solid tumors and enhance the antitumor effect of immune cells. This indicates that OV therapy, as a treatment method with good safety and continuously improved efficacy, may become a powerful supplement to classical therapy, and even produce disruptive results that could change the current treatment method for solid tumors.

## Author contributions

The author confirms being the sole contributor of this work and has approved it for publication.
